# A Multisensor Data Fusion Method Based on Gaussian Process Model for Precision Measurement of Complex Surfaces

**DOI:** 10.3390/s20010278

**Published:** 2020-01-03

**Authors:** Ji Ding, Qiang Liu, Mingxuan Bai, Pengpeng Sun

**Affiliations:** 1 School of Mechanical Engineering & Automation, Beihang University, Beijing 100083, China; 2 Beijing Engineering Technological Research Center of High-efficient & Green CNC Machining Process and Equipment, Beijing 100083, China

**Keywords:** data fusion, data registration, adaptive distance function, complex surface measurement, Gaussian process model

## Abstract

As multisensor measurement technology is rapidly applied in industrial production, one key issue is the data fusion procedure by combining several datasets from multiple sensors to obtain the overall geometric measurement. In this paper, a multisensor data fusion method based on a Gaussian process model is proposed for complex surface measurements. A robust surface registration method based on the adaptive distance function is firstly used to unify the coordinate systems of different measurement datasets. By introducing an adjustment model, the residuals between several independent datasets from different sensors are then approximated to construct a Gaussian process model-based data fusion system. The proposed method is verified through both simulation verification and actual experiments, indicating that the proposed method can fuse multisensor measurement datasets with better fusion accuracy and faster computational efficiency compared to the existing method.

## 1. Introduction

With the development of advanced manufacturing technology, many complex surfaces such as freeform surfaces and structured surfaces can be machined with high precision [1,2]. These surfaces require a complete 3D characterization, with a large measurement range, high resolution and precision, and high measurement efficiency, which poses a challenge to current measurement technology [3,4].

Currently, measuring instruments have been developed to meet specific measuring requirements. Although these instruments have their advantages, no single instrument can simultaneously meet the high requirements in terms of precision, efficiency, resolution, and measuring range [5,6,7]. For example, coordinate measuring machines (CMMs) usually have high accuracy, but their measurement efficiency is low. Non-contact measuring devices, e.g., structured light scanners, could generate dense measurement data efficiently, which can capture the overall shape of the product well. Yet their measurement accuracy is much lower than the CMMs. Therefore, the combination of multiple measurement sensors could be a better solution to solve complex measurement tasks, which maximizes the advantages of individual measurement sensors [8,9,10]. By integrating multiple sensors, these instruments can provide multiple measurement results according to the user’s choice and adapt to the complexity of product geometry measurement. For example, the contact-type CMMs can be optionally equipped with corresponding non-contact measuring sensors. Data fusion is a key problem following multisensor measurement, which can be used in quality inspection, surface reconstruction, and other fields. The aim of data fusion is to integrate multiple datasets from different sources into a unified and improved output. The quality of this output data depends heavily on the data fusion method. Multisensor datasets are usually in different coordinate systems with different measurement ranges, accuracy, and resolution, which brings great challenges to multisensor data fusion [11,12].

In most situations, the multisensor data fusion process is divided into three parts: pre-processing, data registration, and data fusion [4]. Data registration and fusion is the key to realize the whole process. In the data registration, multisensor datasets in different reference coordinate systems are unified into a common coordinate system by finding a rigid body transformation matrix. The Iterative Closest Point (ICP) algorithm proposed by Besl and McKay [13] is the most widely used surface registration algorithm. Its key contribution is to put forward an iterative registration method. The method firstly finds the closest point, and then establishes an optimization model to minimize the error function, thus obtaining updated transformation parameters. When the iteration number reaches a given value, or the error reaches a threshold, the iteration process stops. However, the point-point distance function adopted by ICP has linear convergence according to the literature [14], so ICP and related algorithms have a slow convergence rate. The squared distance minimization (SDM) and tangent-squared distance minimization (TDM) are introduced, which both are the point-tangent distance functions and converge faster than ICP [14,15,16]. However, they need a good initial value of rigid body transformation, otherwise, it is easy for the registration to fail. So, the adaptive distance function (ADF), which is a point-surface distance function, is proposed [17,18]. The ADF maintains a fast convergence rate and has a low requirement for the initial value and good convergence stability [17].

The fusion step mainly deals with the multisensor datasets in a common reference coordinate system after registration, and research on this issue is still inadequate. Jamshidi et al. [19] proposed a high-resolution and low-resolution data fusion method, which realized data fusion by eliminating outliers and gridding all data points after the data registration. However, such methods only substitute the data from different sources locally and cannot improve the accuracy of the overall data. Therefore, the data fusion methods based on modern statistical theory have received much attention [20,21,22,23]. The core idea of such methods is to use statistical methods to model the measurement datasets obtained from different sensors, and then provide the final prediction of each position. Senin et al. [24] compared three multisensor point set augmentation methods, including linear interpolation, locally weighted scatterplot smoothing (LOWESS), and a Gaussian process (GP). Ren et al. [25] presented a weighted least squares based fusion method for precision measurement of free surfaces, which used B-spline surface to fit a linear surface model of different sensor datasets. This method depends on the accuracy of the surface model fitting, and is not suitable for several complex surfaces. Xia et al. [26] introduced a fusion algorithm for high and low accuracy point cloud, which is based on the Bayesian Hierarchical (BH) model. This method could establish a multi-layer Bayesian parameter estimation model for corresponding points of different datasets, and obtain the optimal estimation value after fusion by solving the BH model. Colosimo et al. [27,28,29] proposed a multisensor data fusion method based on the GP model. Reconstruction models of high-accuracy datasets and low-accuracy datasets were established by using the GP model to obtain the optimized fusion dataset. However, these methods rarely consider the effect of different registration methods on fusion quality.

In this paper, a multisensor data fusion algorithm based on GP is presented for precision measurement of freeform surfaces. It can be applied to the data fusion process of 3D point clouds obtained from the same complex surface by multiple sensors. The proposed algorithm utilizes a robust registration algorithm, which is based on the ADF, to unify different coordinate systems, and then uses the GP model to combine multiple datasets from different sensors. The main content of the rest in this paper is divided into three sections. The optimization model and its solution of the ADF based robust registration method is proposed in the second section, and then a GP-based data fusion system is established to generate a surface fusion dataset. The third section gives the verification results of the proposed method under simulation and the actual measurements. The conclusions are presented in the fourth section.

## 2. The Multisensor Data Fusion Method

### 2.1. Summary of the Multisensor Fusion Method

Datasets measured by multisensor systems generally have different resolutions and uncertainties, and they are in different coordinate systems. The main goal of this study is to improve local trueness of the dataset (reduce local bias) by integrating the dataset obtained by the structural light scanner or similar high-speed measurement technology (high point density, low accuracy, low cost) with the dataset obtained by the “more real” point obtained by a CMM or similar technology (slow sampling speed, high accuracy, high price). Therefore, it is assumed that there are two datasets available in this data fusion method:

- one type of dataset with high accuracy, low density, which is generated by CMM or high-precision microscope. This high-accuracy dataset is called the HA set for short.
- another type of dataset with low accuracy, high density, generated by the structured light scanner, line scanner, or similar technology. This low-accuracy dataset is referred to as the LA set.

**Figure 1:**
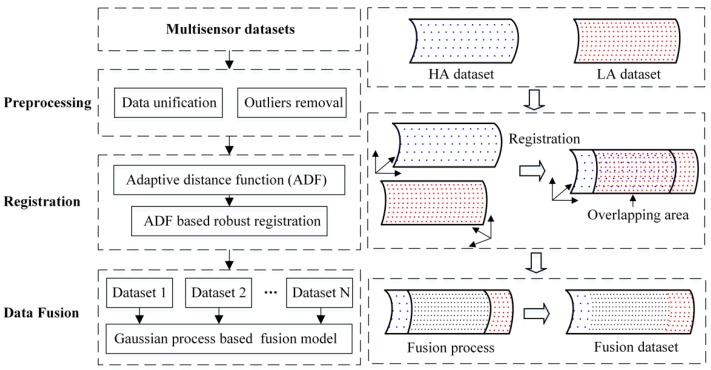
The flow diagram of the multisensor data fusion method.

In order to solve the key problems in data fusion, this paper puts forward a method that divides the whole fusion process into preprocessing, data registration, and data fusion. The flow diagram of the proposed data fusion method is illustrated in Figure 1. The first step is to preprocess the HA and LA data, including data unification, outliers removal, and other operations. The second step is to introduce a robust registration method based on the ADF to unify the coordinate systems of HA and LA datasets. The third step is to establish a GP based multi-layer fusion model to realize the fusion process, in which HA data points are taken as reference points so as to correct the local deviation of the LA dataset.

### 2.2. ADF-Based Robust Data Registration

In this study, the ADF is used to describe the basis error metric, and the M-estimation is used to improve the registration robustness and eliminate noise effects.

**Figure 2:**
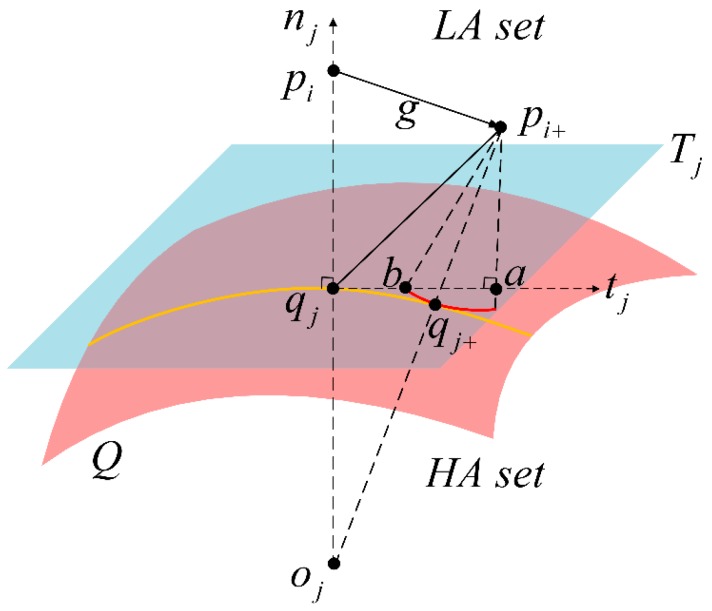
The distance $p_{i+}q_{j+}$ between point $p_{i+}$ and point set Q.

As is shown in Figure 2, we refer to the point set $P=\left\{p_{i}:i=1,\cdots ,n\right\}$ as the LA dataset and the point set $Q=\left\{q_{j}:j=1,\cdots ,m\right\}$ as the HA dataset. The robust registration of point clouds in ℝ^3^ is to find a rigid body transformation g=(R,t)∈ℝ^3^ (including the rotation matrix R ∈ ℝ^3^ and translation matrix t ∈ ℝ^3^) that fits the mobile point set P to the reference point set Q. Point $p_{i+}$ is generated from point $p_{i}$ through rigid body transformation g. Point $q_{j}$ is the nearest to point $p_{i}$, and the tangent plane at point $q_{j}$ is called the plane $T_{j}$. The point $q_{j+}$ at point set Q is obtained in the line $p_{i+}o_{j}$ where the point $o_{j}$ is the center of the curvature circle at $q_{j}$. The normal vector to the surface point set Q at point $q_{j}$ is denoted by the notation $n_{j}$.

It is notable that $\left\|p_{i+}q_{j+}\right\|$ is an approximate expression of the shortest distance between point $p_{i}$ and surface Q. However, the distance $\left\|p_{i+}q_{j+}\right\|$ depends on the curvature change in the region around point $q_{j}$, and the direct calculation of $\left\|p_{i+}q_{j+}\right\|$ is complicated and susceptible to noise. To calculate the transformation g, a new expression for the shortest distance is studied. Point a is obtained by projecting point $p_{i+}$ onto plane $T_{j}$. The arc $bq_{j+}$ is generated by the center $p_{i+}$ and the radius $\left\|p_{i+}q_{j+}\right\|$, then $\left\|p_{i+}q_{j+}\right\|=\left\|p_{i+}b\right\|=\sqrt{{\left\|p_{i+}a\right\|}^{2}+{\left\|ab\right\|}^{2}}$. The tangent distance ‖ab‖ is calculated by ${\left\|ab\right\|}^{2}=\mu {\left\|aq_{j}\right\|}^{2}(\mu \in [0,1])$, where μ denotes the modified coefficient. Then, the ADF is defined as

(1) $$\begin{array}{ll} d_{ADF}^{}\left(g,Q\right) & =\left\|p_{i+}b\right\|=\sqrt{{\left\|p_{i+}a\right\|}^{2}+\mu {\left\|aq_{j}\right\|}^{2}}\\ & =\sqrt{{\left\|n_{j}^{T}\left(p_{i+}-q_{j}\right)\right\|}^{2}+\mu {\left\|t_{j}^{T}\left(p_{i+}-q_{j}\right)\right\|}^{2}},\mu \in [0,1] \end{array}$$

where $n_{j}$ represents the normal vector, $t_{j}$ represents a vector parallel to the tangent plane $T_{j}$.

As the modified coefficient μ goes to 0, the expression $d_{ADF}^{}\left(g,Q\right)$ becomes $d_{TDF}^{}\left(g,Q\right)$, which turns into the point-tangent distance function $\left\|p_{i+}a\right\|$ that is applied to the TDM algorithm. As the modified coefficient μ approaches 1, the expression of ADF becomes $d_{PDF}^{}\left(g,Q\right)$, which corresponds to the point-point distance function $\left\|p_{i+}q_{j}\right\|$ used in the ICP algorithm. Thus, $d_{TDF}^{}\left(g,Q\right)$ and $d_{PDF}^{}\left(g,Q\right)$ both are the special forms of the adaptive function distance (ADF). The ADF selects the appropriate modified coefficient to calculate the tangent distance, so as to reflect the curvature characteristics. In general, the ADF can describe the shortest point-surface distance more accurately than TDM and ICP [17,18].

In order to calculate the rigid body transformation g, an objective function for ADF-based robust registration is established:

(2) $$f\left(g\right)=\sum_{i=1}^{n}\rho \left(d_{ADF}^{}\left(g\left(p_{i}\right),q_{i}\right)\right)$$

where ρ denotes an M-estimate function, $d_{ADF}^{}$ represents the ADF, and the corresponding pairs $\left(p_{i},q_{i}\right)$ are selected from the registration point sets. The M-estimate function ρ has the property of reducing the impact of the data points with large deviation. The derivative of the M-estimate function ρ is written as $\psi \left(e\right)=\rho^{\prime }\left(e\right)$, and a weight function ω is represented as $\omega (e)=\frac{\psi (e)}{e}$.

The bisquare estimator is a typical example of the M-estimators. The bisquare estimator has the strongest suppression ability to the high impact of noisy data. The weight function for the Bisquare estimator is as follow:

(3) $$\omega \left(e\right)=\{\begin{array}{cc} {\left[1-{\left(\frac{e}{k}\right)}^{2}\right]}^{2} & \;\;for\left|e\right|\leq k\\ 0 & for\left|e\right|>k \end{array}$$

In order to solve the objective function for ADF-based robust registration, the objective function with the Bisquare estimator can be transformed into iterative reweighted least squares (IRLS) minimization, and then a new objective function IRLS-ADF is expressed as:

(4) $$f\left(g\right)=\sum_{i=1}^{n}\omega_{i}d_{ADF}^{2}\left(g\left(p_{i}\right),q_{i}\right)$$

with the weight function ω for the Bisquare estimator being a function of the corresponding pairs $\left(p_{i},q_{i}\right)$.

To minimize the objective function for robust registration, a nonlinear optimization model is established:

(5) $$\min f\left(g\right)=\sum_{i=1}^{n}\omega_{i}d_{ADF}^{2}\left(g\left(p_{i}\right),q_{i}\right)s.t.\;q_{i}=q|\underset{q\in Q}{\min\left\|p_{i}-q\right\|}$$

The transformation can be computed by solving the linear equations, similar to [15].

Define $\nu ={\left[Vx,Vy,Vz\right]}^{T}$ as the translation vector, and $\omega ={\left[\delta x,\delta y,\delta z\right]}^{T}$ as the rotation vector, then $\Delta x=p_{i+}-p_{i}=\nu +\omega \times p_{i}$. Define $\xi ={\left[\nu ,\omega \right]}^{T}$, then

(6) $$\begin{array}{l} p_{i+}b=d_{i1}+n_{j}^{T}(\nu +\omega \times p_{i})=d_{i1}+A_{i}\xi \\ bq_{j}=d_{i2}+t_{j}^{T}(\nu +\omega \times p_{i})=d_{i2}+B_{i}\xi \end{array}$$

where $A_{i}$ is a 1 × 6 matrix and $\xi ={\left[\nu ,\omega \right]}^{T}$ is a 6 × 1 matrix.

Therefore, the objective function in (5) can be written as

(7) $$\begin{array}{ll} \varepsilon (\xi ): & =\sum_{i}^{n}\left\{\omega_{i}{\left[d_{i1}+n_{j}^{T}(\nu +\omega \times p_{i})\right]}^{2}+\omega_{i}\mu {\left[d_{i2}+t_{j}^{T}(\nu +\omega \times p_{i})\right]}^{2}\right\}\\ & =D+2C^{T}\xi +\xi^{T}A\xi +\mu \xi^{T}B\xi \end{array}$$

where $A=\sum_{i}^{n}\omega_{i}A_{i}{}^{T}A_{i}$ and $B=\sum_{i}^{n}\omega_{i}B_{i}{}^{T}B_{i}$ are both 6 × 6 symmetric matrices, $C=\sum_{i}^{n}\omega_{i}d_{i}A_{i}{}^{T}$ is a column vector, and D is a scalar.

The parameter $\xi ={\left[\nu ,\omega \right]}^{T}$ is obtained from the solution to the linear equations

(8) (A+μB)ξ+C=0

Then, we can get the optimal transformation g=(R,t) from

(9) $$R=e^{\hat{\omega }},t=\nu$$

where $\hat{\omega }$ is the anti-symmetric matrix of the rotation vector ω.

**Figure 3:**
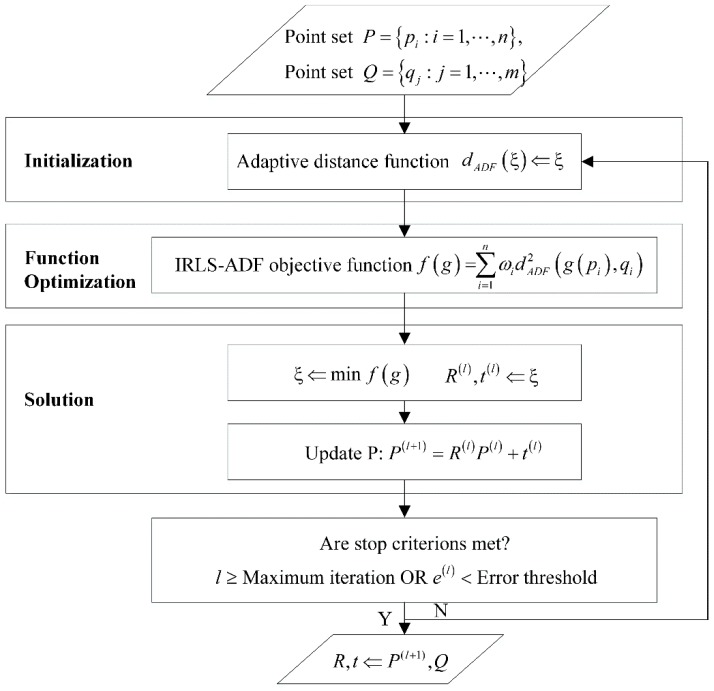
The flow chart of the IRLS-ADF method.

The flow chart of the IRLS-ADF method is given in Figure 3.

In order to comprehensively consider the convergence speed and stability of the IRLS-ADF method, the modified coefficient μ needs to be carefully selected [17,18]. When the iterative process begins, the optimization problem, which becomes a large residual problem, needs to select a large modified coefficient (μ_0_ ∈ [0.5, 0.8]) to keep a certain ratio of tangent distance in the objective function, thus guaranteeing the initial convergence stability. After a certain number of iterations, the optimization problem needs to select a relatively small modified coefficient μ to speed up the convergence rate, because it becomes a small residual problem.

It is proved that the IRLS-ADF method converges to the global minimum for the Bisquare estimator, when the appropriate transformation parameters are selected at the beginning [30]. In fact, the IRLS-ADF method is a kind of Levenberg–Marquardt method for solving the large residual problem, which has good convergence stability and quadratic convergence rate [17,30].

### 2.3. GP-Based Data Fusion Method

After registration, a registered LA dataset and HA dataset are integrated into an improved output by a multi-layer Bayesian data fusion method based on GP model. The fusion method mainly consists of two steps: (1) obtaining an alternative model of the LA dataset; (2) introducing the adjustment model to combine LA and HA datasets together.

Assume that the two datasets can be expressed by discrete functions of the type: $z(x_{i},y_{i})$, that is the *z*-coordinate of the *i*-th point is represented as a function of its position on the *x*,*y* plane. Then, the LA dataset is denoted as: $z_{LA}(x_{i},y_{i})$, where $(x_{i},y_{i})\in V_{LA}\subset$ℝ^2^, i = 1, 2, …, n_LA_ and similarly, the HA dataset is denoted as: $z_{HA}(x_{i},y_{i})$, where $(x_{i},y_{i})\in V_{HA}\subset$ℝ^2^, i = 1, 2, …, n_HA_. Generally, the points in LA and HA dataset will not have the exact same position on the plane, i.e., $V_{LA}\cap V_{HA}=\emptyset$.

The LA data $z_{LA}(x_{i},y_{i})$ can be defined as

(10) $$z_{LA}(x_{i},y_{i})=f_{LA}(x_{i},y_{i})+\varepsilon_{LA}$$

where, $\varepsilon_{LA}$ represents the error, and it is assumed to follow a normal distribution of 0 mean and $\sigma_{\varepsilon LA}^{2}$ variance, i.e., $\varepsilon_{LA}\sim N(0,\sigma_{\varepsilon LA}^{2})$. In an ideal situation, $f_{LA}(x_{i},y_{i})$ represents the ideal value of the measured surface and $\varepsilon_{LA}$ represents the measurement error of the LA dataset. In this paper, a GP model is considered to represent $f_{LA}(x_{i},y_{i})$. The GP model is a set of random variables, in which any finite number of random variables and their linear combinations obey the joint Gaussian distribution. Therefore, according to the GP model, it can be expressed as follows:

(11) $$f_{LA}(x_{i},y_{i})\sim GP(m_{zLA}(v_{i}),k_{zLA}(v_{i},v_{j}))$$

where $v_{i}$ represents $\left(x_{i},y_{i}\right)$ and similarly, $v_{j}$ represents $\left(x_{j},y_{j}\right)$, $m_{zLA}(v_{i})=E\left[z_{LA}(v_{i})\right]$ is the mean function, $k_{zLA}(v_{i},v_{j})=\mathrm{cov}\left(z_{LA}(v_{i}),z_{LA}(v_{j})\right)$ is the covariance function. In this paper, the mean function can be expressed with a linear model:

(12) $$m_{zLA}(x_{i},y_{i})=\alpha_{0}+\alpha_{1}x_{i}+\alpha_{2}y_{i}$$

In addition, the square exponential function is chosen to represent the covariance function of the GP model:

(13) $$k_{zLA}(v_{i},v_{j})=\sigma_{zLA}^{2}\exp(-\frac{{\left\|v_{i}-v_{j}\right\|}^{2}}{2l_{LA}^{2}})$$

where $\left\|v_{i}-v_{j}\right\|$ represents the Euclidean distance between vector $v_{i}$ and vector $v_{j}$, $\sigma_{zLA}^{2}$ represents the constant variance of the GP model, and $l_{LA}^{}$ is the length parameter of the function.

In the model expressed by Equations (10)–(13), parameters $\left\{\alpha_{0},\alpha_{1},\alpha_{2},\sigma_{zLA}^{2},l_{LA}^{},\sigma_{\varepsilon LA}^{2}\right\}$ are unknown and need to be estimated by actual measurement data. Therefore, as long as the measurement data $z_{LA}(x_{i},y_{i})$ with $(x_{i},y_{i})\in V_{LA}\subset$ℝ^2^, i = 1, 2, …, n_LA_ is given, the new prediction value ${\hat{z}}_{LA}(x,y)$ can be computed at any new position (x,y) according to the mean and covariance functions after the completion of the parameter estimation.

However, the alternative model based on the LA data cannot correct the possible local bias in the LA dataset. Due to the high accuracy and unbiasedness of the HA dataset within the measurement range, we introduce the high-quality data of the HA dataset to “correct” the alternative model generated by the LA dataset. In order to describe the difference between the values ${\hat{z}}_{LA}(x_{i},y_{i})$ obtained by the alternative model generated from LA dataset and the values of HA dataset $z_{HA}(x_{i},y_{i})$, the “adjustment model” is introduced as in literature [26,27]:

(14) $$z_{HA}(x_{i},y_{i})=\beta (x_{i},y_{i}){\hat{z}}_{LA}(x_{i},y_{i})+\delta (x_{i},y_{i})+\varepsilon$$

where $\beta (x_{i},y_{i})$ and $\delta (x_{i},y_{i})$ represent the scaling factor and the shifting factor, respectively, and ε represents the error term. The adjustment model means that, for positions containing HA points, the value $z_{HA}(x_{i},y_{i})$ can be obtained by scaling and shifting the predicted value ${\hat{z}}_{LA}(x_{i},y_{i})$ estimated by the GP model from the LA dataset, plus an error item associated with the HA dataset. Since the adjustment model is statistically established, it should contain the error term ε to indicate residuals between the HA dataset $z_{HA}(x_{i},y_{i})$ and $\beta (x_{i},y_{i}){\hat{z}}_{LA}(x_{i},y_{i})+\delta (x_{i},y_{i})$. Assume that the residual term ε obeys a normal distribution $\varepsilon \sim N(0,\sigma_{\varepsilon }^{2})$.

The scaling factor β can be expressed by the linear model:

(15) $$\beta (x_{i},y_{i})=\beta_{0}+\beta_{1}x_{i}+\beta_{2}y_{i}$$

The shifting factor δ can be represented by a new GP model composed of the constant mean value $\delta_{0}$ and covariance function $k_{\delta }$:

(16) $$\delta (x_{i},y_{i})\sim GP(\delta_{0},k_{\delta })$$

where the square exponential function is used to represent the covariance function $k_{\delta }=\sigma_{\delta }^{2}\exp(-\frac{{\left\|v_{i}-v_{j}\right\|}^{2}}{2l_{\delta }^{2}})$, as the same in Equation (13).

The parameters $\left\{\beta_{0},\beta_{1},\beta_{2},\delta_{0},\sigma_{\delta }^{2},l_{\delta }^{},\sigma_{\varepsilon }^{2}\right\}$ in Equations (14)–(16) are all unknown and can be estimated from the actual measurement data $z_{HA}(x_{i},y_{i})$ with $(x_{i},y_{i})\in V_{HA}\subset$ℝ^2^, i = 1, 2, …, n_HA_.

After fully estimating all the unknown parameters mentioned above, correction parameters based on the HA dataset (unbiased) can be obtained according to the adjustment model. Therefore, the original GP model (biased) prediction data can be modified to

(17) $$z_{fusion}(x_{i},y_{i})=\beta (x_{i},y_{i}){\hat{z}}_{LA}(x_{i},y_{i})+\delta (x_{i},y_{i})$$

## 3. Experimental Verification

### 3.1. Simulation Verification

**Figure 4:**
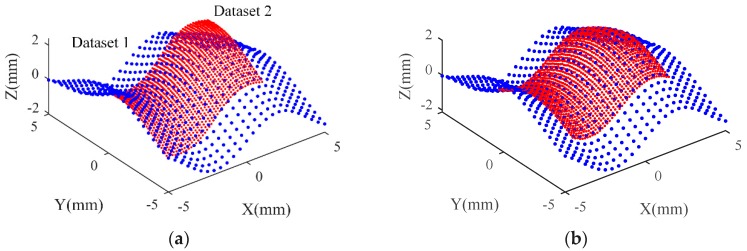
Multisensor data for simulation and data registration results. (**a**) Two simulation datasets, where blue data represents Dataset 1 and red data represents Dataset 2; (**b**) Data registration results based on IRLS-ADF method.

In order to analyze the performance of the proposed data fusion algorithm, a freeform surface is defined by Equation (18):

(18) z=sin(0.8x)+cos(0.5y)

where x,y∈[−5,5] mm. On such a surface, a set of points is sampled uniformly across the whole surface at an interval of 0.4 mm, which is defined as Dataset 1. Another set of points is sampled at 0.15 mm interval over a part x,y∈[−3,3] of the surface, which is denoted as Dataset 2. Dataset 1 represents the HA dataset, Dataset 2 is equivalent to the LA dataset, and two sets of Gaussian noise with standard deviation 5 μm and 15 μm are added to Dataset 1 and Dataset 2, respectively, to represent measurement errors of the HA and LA datasets. Dataset 2 is moved to a specific location so that the two sets of points are located in different coordinate systems. The given transformation parameters are $t_{x}=1\;mm,t_{y}=1\;mm,t_{z}=-0.5\;mm$ (translation parameters) and $r_{x}=-0.1\;rad,r_{y}=0.3\;rad,r_{z}=0.2\;rad$ (rotation parameters). Therefore, these two sets of point cloud data have different resolutions, different uncertainties, and different coordinate systems. These two datasets are common in the multisensor measurement of the freeform surface. The generated Dataset 1 and 2 systems are shown in a common coordinate in Figure 4a.

**Figure 5:**
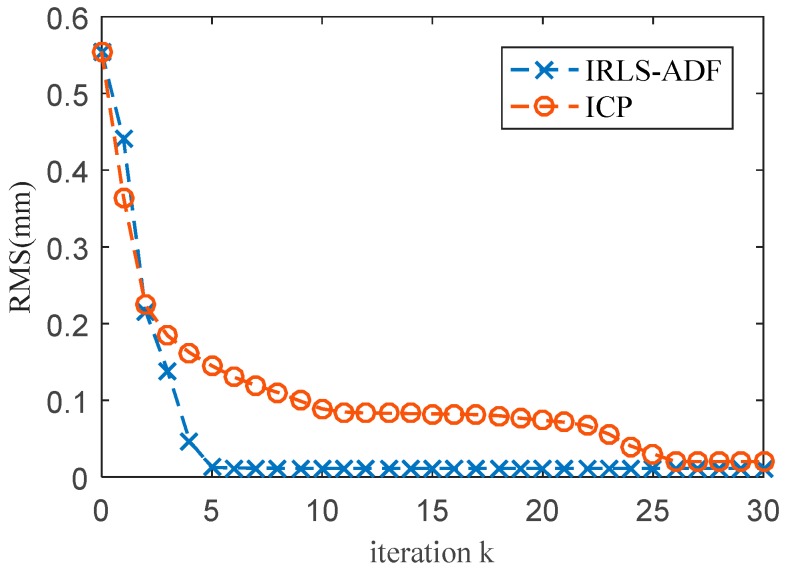
Comparison of registration errors between the IRLS-ADF and ICP methods during the iteration process.

Firstly, the proposed robust registration algorithm based on ADF is used to register Dataset 1 and Dataset 2, and unify them into a common coordinate system. Figure 4b shows the data registration results based on IRLS-ADF. In order to evaluate the accuracy and efficiency of the proposed algorithm, the transformation parameter error and calculation time of the IRLS-ADF and ICP methods are presented in Table 1. Figure 5 shows a comparison of the registration errors between the IRLS-ADF and ICP methods during the iteration process. Both methods are implemented using MATLAB R2016a, with a maximum of 30 iterations, and run on a computer with the Intel^®^ Core^TM^ i7(8 GB RAM) processor. The transformation parameter errors of the IRLS-ADF method is smaller than the ICP method, which indicates that the IRLS-ADF method has higher accuracy. The iteration number of the IRLS-ADF is significantly smaller than that of the ICP method, and the calculation time of IRLS-ADF is also shorter because it is based on the Levenberg–Marquardt method, and achieves faster convergence rates. This result is consistent with the literature [17]. Generally, the IRLS-ADF method has higher precision and faster convergence rates for HA and LA dataset registration, indicating its robustness against the influence of the measurement error.

**Figure 6:**
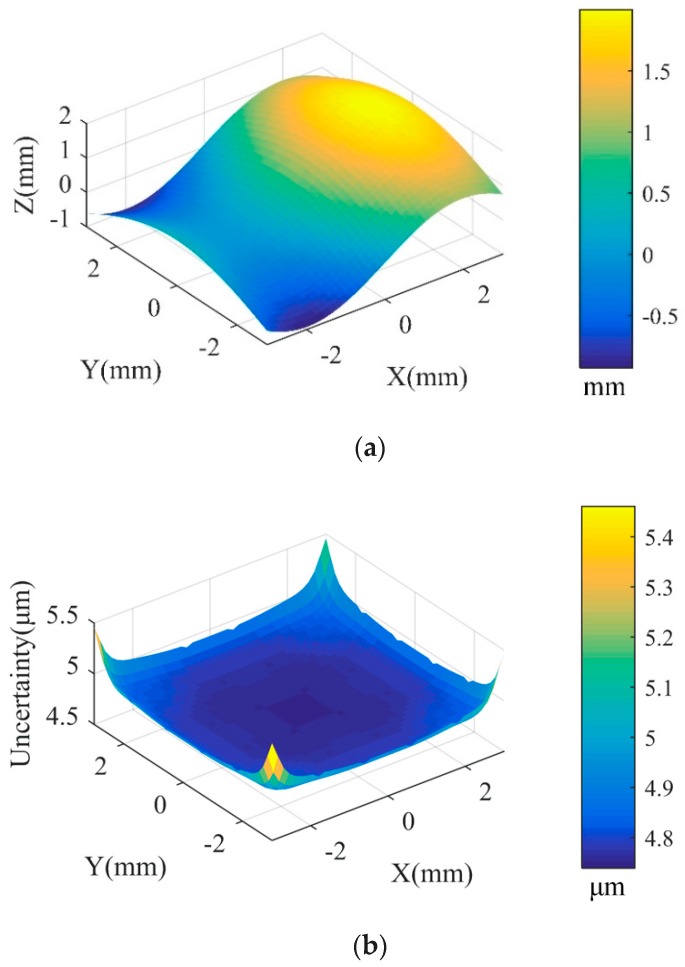
(**a**) The fusion result of the overlapping area based on the GP model; (**b**) Uncertainty estimated by the established GP fusion model.

**Figure 7:**
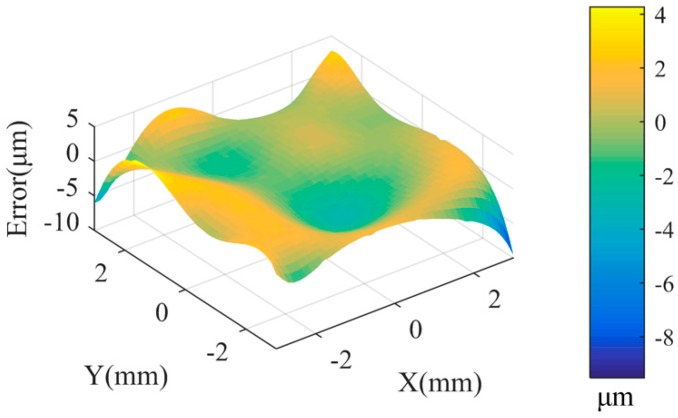
Evaluated error diagram of the fusion data.

Then, a data fusion model based on the GP model is constructed in the overlapping area according to Section 2.3. Figure 6 shows the fusion data and its estimation uncertainty. Figure 7 shows the form error of the fusion data by comparing it with the designed surface. In order to evaluate the accuracy of the fusion data, the root-mean-square (RMS) error and peak-to-valley (PV) error of the reconstructed model data compared with the designed surface are calculated. Table 2 shows the RMS and PV error comparison between Dataset 1, Dataset 2, and the proposed IRLS-ADF+GP fusion method. It can be seen that the RMS values of Datasets 1 and 2 are 4.3 μm and 14.9 μm, respectively, which is consistent with the measurement noise added in these datasets. Based on the proposed data fusion method, the RMS value of the fusion data is reduced to 1.9 μm, which is significantly lower than the RMS values of Dataset 1 and 2. Similarly, the PV value of the fused data is reduced to 13.8 μm. It shows that the proposed GP-based fusion method is capable of improving the accuracy of the measured datasets. The proposed method is also compared with the widely used data fusion method, which adopts the ICP registration method to align two datasets and integrates them with the weighted mean (WM) method [3]. It is also seen from the results of Table 2 that the proposed method has better fusion accuracy and faster computational efficiency than the ICP-WM method.

**Figure 8:**
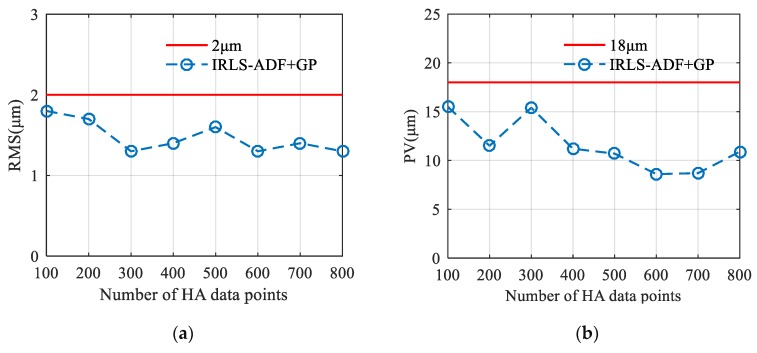
Final RMS and PV values of fusion results when the number of the HA point changes to N={100,200,300,400,500,600,700,800}, respectively. (**a**) RMS error; (**b**) PV error.

In addition to data fusion performance, measurement efficiency is also a key concern in the multisensor measurement. LA datasets can be obtained through a structured light scanner with high measurement efficiency. However, HA datasets are usually acquired by CMMs, and the measurement efficiency is low. Only if the number of HA data is small, the measurement time is acceptable. Therefore, multiple datasets (still called Dataset 1), which contain a different number of data points in the overlapping area, are used to evaluate the performance of the proposed algorithm fully. A set of Gaussian noise with standard deviation 5 μm is still added to Dataset 1, and Dataset 2, which is used for fusion with Dataset 1, remains unchanged. Figure 8 shows the final RMS and PV values of fusion results generated by the proposed IRLS-ADF+GP fusion method with the change of HA point number in Dataset 1. As can be seen from Figure 8a, the RMS values of the fusion data do not change much with the decrease of the HA point number in Dataset 1, and remain at a low level (<2 μm). Similarly, as the number of data points in Dataset 1 decreases, the PV values of the fusion data remains low (<18 μm), as shown in Figure 8b. It indicates that the proposed data fusion algorithm can achieve high-quality data fusion performance with a small number of HA data. In actual multisensor measurements, the efficiency of the multisensor measurement can be significantly improved with few HA data points. Therefore, with the LA dataset and a small amount of HA data points, the data fusion algorithm proposed in this paper can complete the data fusion process with a good combination of measurement efficiency and fusion performance. It should be emphasized that too few HA data points are not recommended for multisensor fusion process (for example, <50 in this experiment), because this means that too few “real points” are used to modify the LA dataset, which may lead to a decrease in the registration accuracy and result in poor data fusion performance.

### 3.2. Verification in Actual Measurement

**Figure 9:**
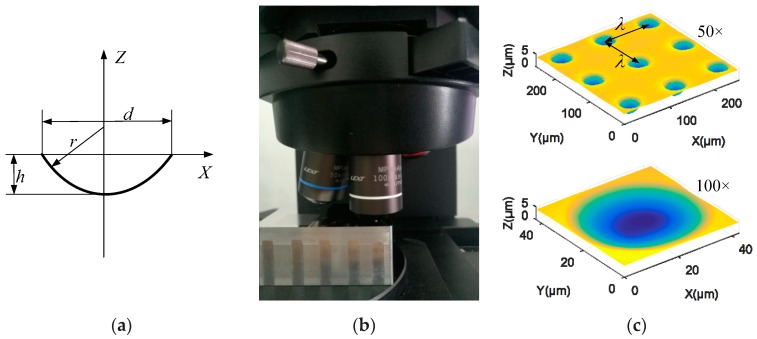
Measurement of a spherical microstructure surface. (**a**) 2D diagram of a single spherical surface; (**b**) The measurement process of the machined surface on a 3D laser microscope; (**c**) Measurement data obtained by 50× and 100× objectives, respectively.

The first example of actual measurement is a spherical microstructure surface. The 2D diagram of a single spherical surface is shown in Figure 9a, where the radius of the single spherical surface is r=52 μm, the depth is h=4 μm, the diameter of the aperture is d=40 μm. The spherical microstructure array is distributed on a square grid, and the spacing between the adjacent lines in both the X and Y direction is λ=100 μm. The above microstructure surface has been machined by an ultra-precision machine tool, and the surface topography is captured by a 3D laser microscope Olympus OLS5000, as shown in Figure 9b. The complete geometric information is measured in two steps with 50× objective (zoom is 1.0×) and 100× objective (zoom is 3.0×), respectively. The field of view of the two objectives is 0.25 × 0.25 mm^2^ and 0.04 × 0.04 mm^2^ respectively. The measurement data are shown in Figure 9c. Due to two different objectives, the two measured datasets have different resolutions and are in different coordinate systems. Then, the proposed method in this paper is used for the registration and fusion process of the above two datasets to generate a final representation of the microstructure surface.

**Figure 10:**
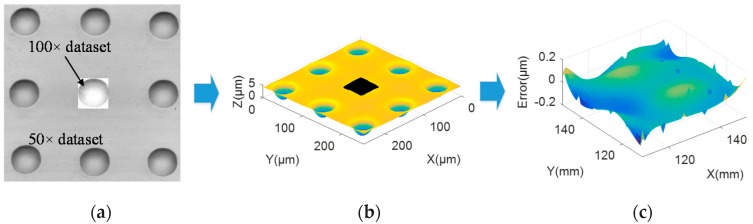
Registration and fusion process of the measured surface data. (**a**) IRLS-ADF registration; (**b**) The registered datasets; (**c**) Evaluated error diagram of the fusion data.

Firstly, the dataset obtained by 100× objective is transformed into the coordinate frame of the dataset measured by 50× objective based on the proposed registration algorithm. Figure 10a shows the registration of measured datasets based on the IRLS-ADF registration method, and Figure 10b presents the datasets after registration. Then, the two datasets at the overlapping area are fused by the proposed fusion method. Finally, the 50× dataset, 100× dataset, and fusion dataset are compared with the original CAD model respectively to verify the quality of the above datasets. The evaluated error diagram of the fusion data is given in Figure 10c. The RMS errors of the three datasets are 0.125 μm, 0.119 μm, and 0.103 μm, respectively. From the above error results, we can see that the dataset processed by the registration and fusion method has higher accuracy than the two original measurement datasets.

**Figure 11:**
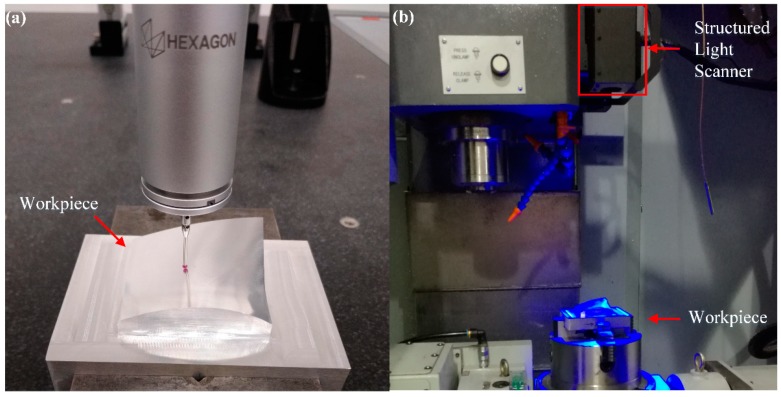
The measurement process of the machined freeform surface. (**a**) The machined freeform surface on the CMM; (**b**) The surface under a structured light (SL) scanner.

The second example of actual measurement is a machined freeform surface with a geometric dimension of 52 mm × 50 mm × 13 mm. The machined surface is measured by two different measurement instruments, namely a high-precision Hexagon Coordinate Measuring Machine (CMM) and a structural light (SL) scanner. Figure 11 shows the measurement process of the machined surface by means of CMM and SL scanner. The length measurement error of the CMM is U= 1.4 + 3.0 × L/1000 μm, and the maximum probing error is MPEP = 1.2 μm. The accuracy of the SL scanner is 0.03 mm, and the field of view is 300 × 240 mm^2^.

**Figure 12:**
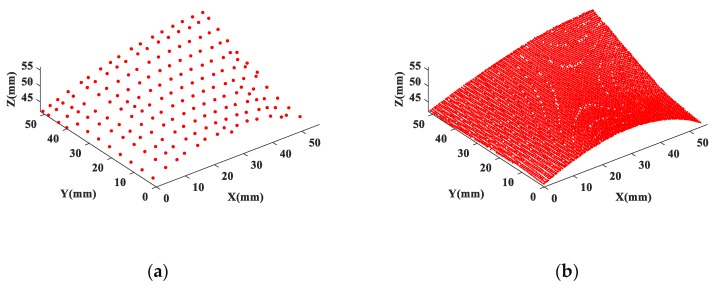
Multisensor measurement datasets. (**a**) CMM measurement data; (**b**) SL measurement data.

Figure 12 shows the measurement datasets obtained from the CMM and the structured light scanner. The CMM dataset is a HA dataset, which consists of $n_{CMM}=165$ points (Figure 12a) with 4 mm spacing in both X and Y direction. The LA dataset consists of $n_{SL}=8131$ SL data points (Figure 12b). The two datasets have different resolutions and uncertainties and are located in different coordinate systems. To verify the fusion performance of the proposed method, we also collect a dataset called a reference dataset, which is composed of $n_{REF}=7131$ CMM data points with 0.6 mm spacing over the measured surface.

**Figure 13:**
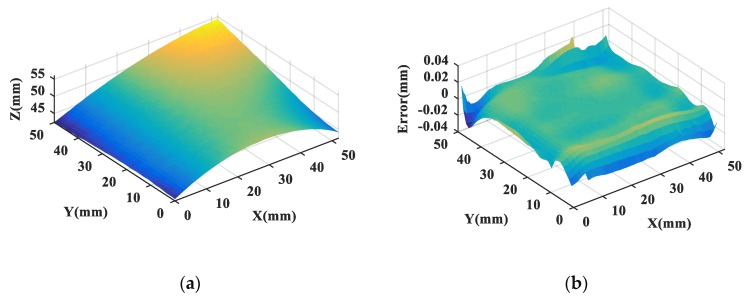
(**a**) Fusion dataset of the measured surface; (**b**) Evaluated error diagram of the fusion data.

The proposed algorithm is applied to the fusion of the above two datasets. IRLS-ADF registration algorithm is used to transform two datasets into a common coordinate system. Then a fusion dataset is generated by the GP-based fusion method. Figure 13a shows the fusion dataset. The evaluated error diagram of the fusion data by comparison with the design surface is, as shown in Figure 13b. In order to analyze the performance of the proposed method, the accuracy of the fusion dataset is characterized by RMS and PV error, and the measurement efficiency of the fusion dataset is reflected by the measurement time. Table 3 shows the comparison results of different measurement data, including the CMM dataset, SL dataset, fusion dataset, and reference dataset.

In terms of accuracy, the RMS value of the fusion dataset generated by the proposed fusion method is 14.1 μm, and the PV value is 66.5 μm. Compared with the SL dataset, the accuracy of the fusion dataset is greatly improved. At the same time, the RMS and PV values of the fusion dataset are slightly worse than that of the reference dataset, indicating that the data quality of the fusion dataset is close to that of the reference dataset. In terms of measurement efficiency, the measurement time of the multisensor fusion method based on CMM and SL data is about 0.4 h, which is far less than the measurement time of the reference dataset. It can be seen from the above results that the accuracy of the fusion dataset is significantly improved by means of registering and fusing datasets from CMM and SL, while the measurement efficiency is maintained. In general, a large number of measurement points in the CMM lead to long sampling time, while few CMM measurement points result in insufficient surface sampling, and the error of the machined surface may be underestimated. And the structured light scanner has a fast sampling speed but relatively low accuracy. Therefore, multisensor combination measurement is of great importance to improve the efficiency and precision of complex surface measurement. The above experimental results indicate that the proposed data fusion method can fuse multisensor datasets of complex geometry, with good fusion accuracy and high measurement efficiency.

## 4. Conclusions

This paper proposes a data fusion method for multisensor combination measurement of complex surfaces. In order to solve the key issues of multisensor fusion, a surface robust registration method is presented to unify the coordinate systems of multisensor datasets, which utilizes the ADF as the basic error metric, and uses M-estimation method to limit the influence of noise points. Then, a GP model-based data fusion method is presented to generate a high-quality fusion dataset. Moreover, the proposed method is verified by simulation and actual experiments. Experimental results show that the proposed method has the capability to fuse multisensor datasets of complex geometry with good fusion performance and fast computational efficiency. In addition, the proposed data fusion algorithm only needs a small amount of HA data points and an LA dataset to complete the data fusion process, achieving a good combination of measurement efficiency and fusion performance.

How to speed up the data fusion method through parallel hardware (FPGA or GPU) is a task that needs to be solved in the future. In addition, the data fusion process may involve more than two datasets in practical application. The proposed data fusion method can only be used for the fusion between two different datasets, and further research is needed. Furthermore, the prior knowledge of the uncertainty associated with each sensor could be utilized in the future research.

## Figures and Tables

**Table 1:** The error of transformation parameters and computation time of the IRLS-ADF and ICP method.

Method	Error of Transformation Parameters						Computation Time (s)
	t_x_ (μm)	t_y_ (μm)	t_z_ (μm)	r_x_ (mrad)	r_y_ (mrad)	r_z_ (mrad)	
IRLS-ADF	1.8	3.7	2.5	0.5	0.2	0.7	1.1
ICP	3.7	5.3	5.2	1.4	5.0	2.1	3.2

**Table 2:** RMS, PV error, and computation time of different data fusion methods.

	Dataset 1	Dataset 2	Fusion by IRLS-ADF+GP	Fusion by ICP+WM
RMS (μm)	4.3	14.9	1.9	3.4
PV (μm)	19.9	57.4	13.8	17
Computation time (s)	-	-	5.5	8.2

**Table 3:** RMS, PV error, and time comparison of different measurement datasets.

Dataset	CMM	SL	Fusion	Reference
RMS (μm)	13.2	17.6	14.1	13.9
PV (μm)	60.4	77.4	66.5	65.4
Time (h)	~0.4	<0.1	~0.4	>2
